# Deep Learning–Powered CT-Less Multitracer Organ Segmentation From PET Images: A Solution for Unreliable CT Segmentation in PET/CT Imaging

**DOI:** 10.1097/RLU.0000000000005685

**Published:** 2025-01-28

**Authors:** Yazdan Salimi, Zahra Mansouri, Isaac Shiri, Ismini Mainta, Habib Zaidi

**Affiliations:** From the ∗ Division of Nuclear Medicine and Molecular Imaging, Geneva University Hospital, Geneva, Switzerland; † Department of Cardiology, Inselspital, Bern University Hospital, University of Bern, Bern, Switzerland; ‡ Department of Nuclear Medicine and Molecular Imaging, University of Groningen, University Medical Center Groningen, Groningen, the Netherlands; § Department of Nuclear Medicine, University of Southern Denmark, Odense, Denmark; ∥ University Research and Innovation Center, Óbuda University, Budapest, Hungary.

**Keywords:** PET/CT, ^68^Ga-PSMA, ^18^F-FDG, organ segmentation, deep learning

## Abstract

**Purpose:**

The common approach for organ segmentation in hybrid imaging relies on coregistered CT (CTAC) images. This method, however, presents several limitations in real clinical workflows where mismatch between PET and CT images are very common. Moreover, low-dose CTAC images have poor quality, thus challenging the segmentation task. Recent advances in CT-less PET imaging further highlight the necessity for an effective PET organ segmentation pipeline that does not rely on CT images. Therefore, the goal of this study was to develop a CT-less multitracer PET segmentation framework.

**Patients and Methods:**

We collected 2062 PET/CT images from multiple scanners. The patients were injected with either ^18^F-FDG (1487) or ^68^Ga-PSMA (575). PET/CT images with any kind of mismatch between PET and CT images were detected through visual assessment and excluded from our study. Multiple organs were delineated on CT components using previously trained in-house developed nnU-Net models. The segmentation masks were resampled to coregistered PET images and used to train 4 different deep learning models using different images as input, including noncorrected PET (PET-NC) and attenuation and scatter-corrected PET (PET-ASC) for ^18^F-FDG (tasks 1 and 2, respectively using 22 organs) and PET-NC and PET-ASC for ^68^Ga tracers (tasks 3 and 4, respectively, using 15 organs). The models’ performance was evaluated in terms of Dice coefficient, Jaccard index, and segment volume difference.

**Results:**

The average Dice coefficient over all organs was 0.81 ± 0.15, 0.82 ± 0.14, 0.77 ± 0.17, and 0.79 ± 0.16 for tasks 1, 2, 3, and 4, respectively. PET-ASC models outperformed PET-NC models (*P* < 0.05) for most of organs. The highest Dice values were achieved for the brain (0.93 to 0.96 in all 4 tasks), whereas the lowest values were achieved for small organs, such as the adrenal glands. The trained models showed robust performance on dynamic noisy images as well.

**Conclusions:**

Deep learning models allow high-performance multiorgan segmentation for 2 popular PET tracers without the use of CT information. These models may tackle the limitations of using CT segmentation in PET/CT image quantification, kinetic modeling, radiomics analysis, dosimetry, or any other tasks that require organ segmentation masks.

PET/CT hybrid imaging provides valuable information by combining structural, molecular, and physiological information with a wide range of indications and radiopharmaceuticals.^1^ Since the emergence of hybrid PET/CT imaging, the development and use of different radiotracers have expanded. ^18^F-FDG, with a wide range of indications for brain, cardiac, and oncological imaging, is the most common used radiotracer in clinical practice.^2–4^ Other common radiotracers are prostate-specific membrane antigen radiolabeled ligands, such as ^68^Ga-PSMA, and radiolabeled somatostatin analogs, such as ^68^Ga-DOTATATE. ^68^Ga-PSMA has high diagnostic accuracy in the initial staging and biochemical recurrence evaluation in patients diagnosed with prostate cancer.^5,6^ It is also used in the context of PSMA radioligand theranostics, for lesions’ evaluation and patient selection with a good predictive value.^7^

Medical image segmentation in general, and in nuclear medicine in particular, is a crucial step toward modern personalized medicine. Segmentation can play an important role in PET image quantification in each organ and volume of interest (VOI).^8,9^ Quantitative PET provides detailed information for accurate diagnosis and therapy by precise measurement of tracer uptakes and kinetics within each VOI.^10,11^ Nowadays, with the growing interest in personalized dosimetry for radiopharmaceutical therapy, aiming at delivering a tumoricidal radiation dose to the target volume while sparing organs at risk,^12,13^ the importance of image segmentation is becoming more pronounced. Moreover, the assessment of treatment response and prognostication, which requires tumor and organs-at-risk masks, have recently received significant attention.^14–18^ In addition, studies highlighted the importance of nontumoral organs in prognostication and outcome prediction.^19,20^

In clinical practice, segmentation is performed manually on CT or MRI scans after visual inspection of PET images.^21–23^ Manual contouring is subjective, time-consuming, labor intensive, and prone to errors and interoperator/intraoperator variability because of different levels of expertise and the use of different windowing settings.^24,25^ The available methods for automated organ segmentation in hybrid molecular imaging, predominantly using deep learning (DL), focus on using coregistered CT images.^26^ Reliable CT segmentation tools capable of automated segmentation^27,28^ can be used for PET/CT images. However, this approach faces 3 main limitations.

First, mismatch between emission (PET/SPECT) and transmission (CT) images is highly prevalent in clinical setting.^29,30^ This issue becomes more challenging in dynamic imaging protocols, where CT images are acquired within seconds at the beginning of the examination, while the dynamic PET scan is usually acquired during much longer time, including inevitably averaging multiple respiratory and cardiac cycles. In addition, involuntary changes in the position and size of the organs, such as the bladder getting filled and bowel movements, limit CT segmentation reliability.^31^ Additionally, patient bulk motion during prolonged dynamic acquisitions further complicate alignment. Second, the low-dose and ultra-low-dose attenuation correction CT (CTAC) images acquired using lower tube currents and special beam filtering,^32^ often used in PET/CT, suffer from reduced image quality, thus affecting the accuracy of segmentation. Last, the potential advent of CT-less clinical scanners, such as PET-only and PET/MRI scanners,^30,33^ which utilize DL-based or MLAA (maximum likelihood estimation of activity and attenuation)–based attenuation correction methods, pose a significant challenge to CT-based segmentation approaches. DL-guided MRI multiorgan segmentation models were recently introduced to overcome PET/MRI organ segmentation.^34^ These limitations highlight the necessity for developing segmentation tools based on emission data only rather than relying on coregistered CT images.

Utilizing the emission data to improve the performance of DL-based organ segmentation has been previously reported.^35–38^ Klyuzhin et al^35^ used both PET and CT images for improved organ segmentation in PET/CT. Yazdani et al^36^ developed DL segmentation models to segment both healthy organs and malignant lesions from ^68^Ga-PSMA PET/CT images and compared them to using only PET, only CT, and both images as input to their DL models. Wang et al^37^ segmented bladder and heart on ^18^F-FDG PET/CT images to overcome the issue of absence or availability of unreliable CT images. Clement et al^38^ developed a model to perform CT-less organ segmentation on ^18^F-FDG PET images for dynamic imaging.

This study aimed to develop a reliable CT-less multiorgan segmentation pipeline on 2 common radiotracers (^18^F-FDG and ^68^Ga-PSMA) using a multicentric dataset to address the limitations of CT-based segmentation approaches in hybrid PET/CT imaging.

## PATIENTS AND METHODS

### Common Processing and Steps for Both Tracers for Reference Segmentation Generation

**FIGURE 1:**
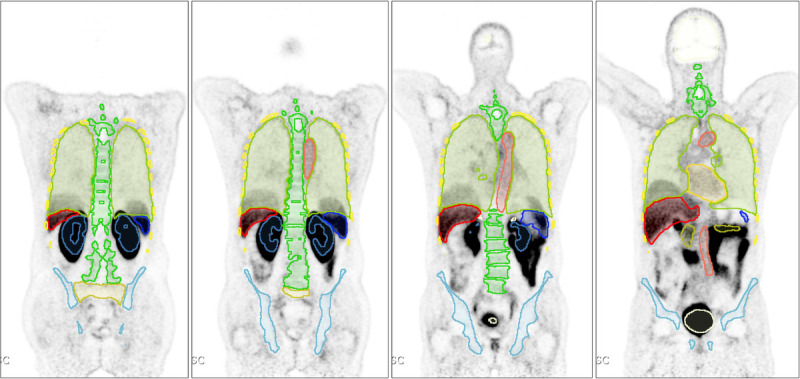
Coronal slices showing representative cases presenting with mismatch between CT-based segmentation and PET images. Please note the chest/abdomen interval organs.

**FIGURE 2:**
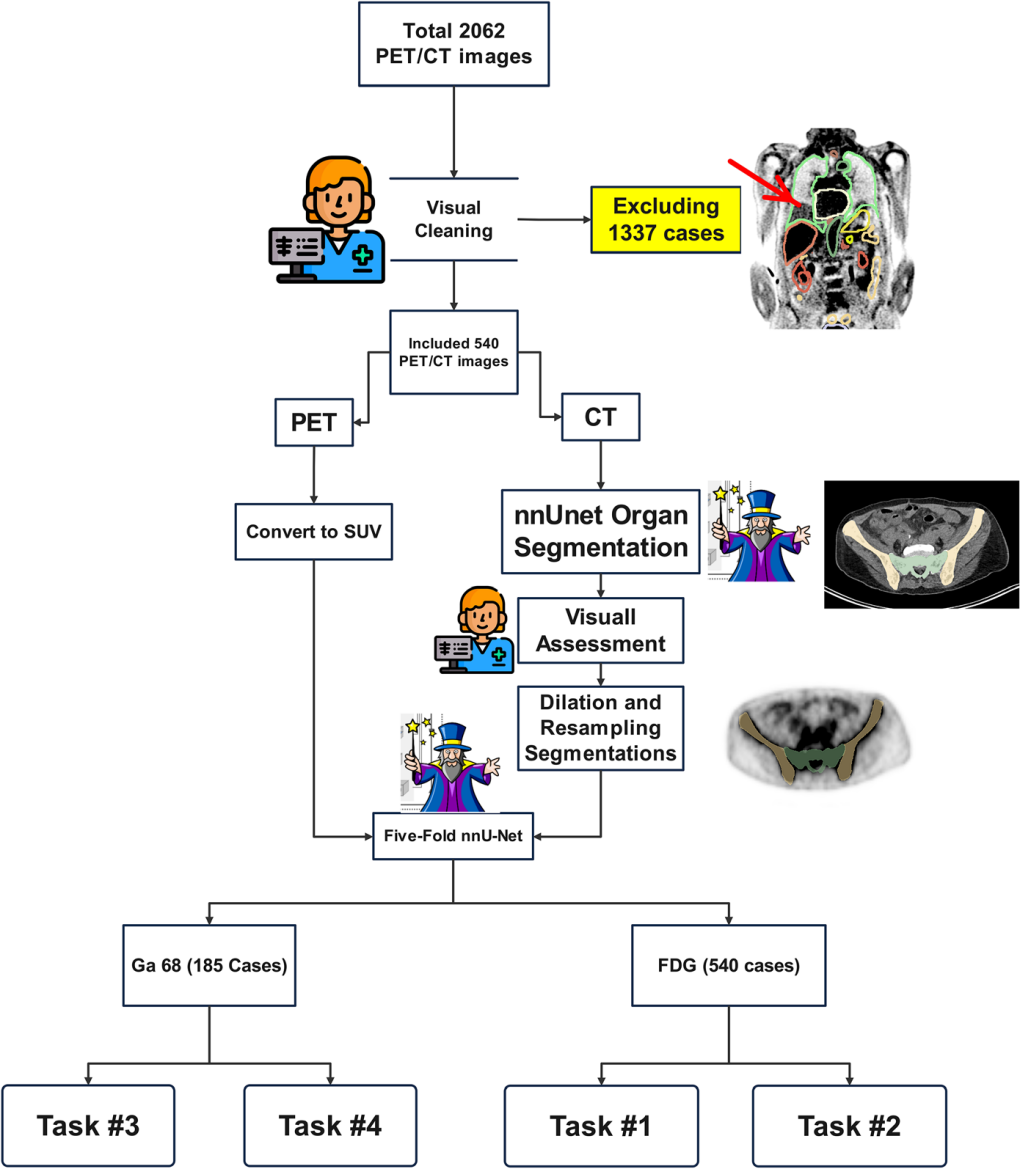
Flowchart of steps followed in this study, including generation of reference segmentation masks and training the LD-nnU-Net models.

Three types of images, including CTAC, noncorrected PET (NC), and attenuation and scatter-corrected (CT-ASC) PET images, were collected in a fully anonymized setup. All images were visualized using the open-source ITK-SNAP software.^39^ Images presenting with a mismatch between PET and CT were excluded from training, whereas PET/CT images without mismatch were included in the next steps. Figure 1 illustrates an example of a mismatch visualized with segmentation generated based on the coregistered CT scan. Using previously developed DL-based segmentation models in our group^28^ based on nnU-Net architecture,^40^ a total number of 22 organs were delineated on the CTAC component of PET/CT images. All nnU-Net 5-folds were assembled on images to ensure the highest segmentation accuracy. The previously trained models were separate models, each dedicated to 1 specific organ. The CT-generated segmentation masks were dilated by 2 mm and resampled with the coregistered PET image voxel spacing. During down sampling from CT voxel spacing (1 to 1.5 mm) to PET spacing (1.6 to 4 mm) without dilation, certain organ shapes, such as ribs and thin parts of pelvic hip bones, may be lost. This occurs because nearest neighborhood interpolation, which is necessary for maintaining a binary segment with values of 0 and 1, has limitations that can result in the removal of fine details in the segmentation. The segmentations were integrated into a unified multivalue segmentation mask using the Simple ITK 2.2 Python library, prioritizing organs with higher Dice values from CT training task. For instance, if a single voxel was segmented as both liver and stomach by separate CT segmentation models, the voxel was classified as liver tissue to avoid overlap between the 2 organs. CT segmentation models consist of 1 independent model per organ, and as such, 2 models may detect single voxels as belonging to different organs. Besides, when a segmentation mask is downsampled from CT to PET resolution, some voxels may be considered as segmentation output of 2 organs with overlap on CT images. For example, when the lung and liver segmentation masks are you downsampled, some voxels may have “1” segment value in both liver and lung areas due to the nature of nearest neighborhood and linear interpolation methods. We prioritized the model with higher performance when making decisions about specific voxels, as we trust the high-performance model more than the low-performance model. The rationale behind this decision is based on the performance metrics and the characteristics of each organ, which determine how easy or difficult a DL model can detect them. In the specific example about the liver and stomach, the CT segmentation model had a Dice coefficient of ~97% and 93% for liver and stomach, respectively. Each voxel needs to be considered as belonging to a single organ for training the multilabel models with no overlap between the organs. Comparing the 2 models’ performance, it is more probable that liver segmentation model is the correct one. The segmentation masks and PET images were used to train an nnU-Net model using a 5-fold cross-validation data split. Four different tasks were defined: 2 utilizing ^18^F-FDG PET images and 2 utilizing ^68^Ga-PET images (Fig. 2). Each task involved using either ASC or NC as inputs, namely, ^18^F-FDG-NC (task 1), ^18^F-FDG-ASC (task 2), ^68^Ga-NC (task 3), and ^68^Ga-ASC (task 4).

### Datasets

This study included 2 separate sets of images acquired from patients injected with ^18^F-FDG (dataset 1) and ^68^Ga-PSMA tracers (dataset 2). The study was approved by the local ethics committee, and consent was waived owing to the retrospective nature of the study protocol.

#### Dataset 1 (^18^F-FDG)

This dataset included patients injected with ^18^F-FDG for oncological indications, with whole-body PET/CT images acquired on Biograph mCT and Biograph Vision scanners (Siemens Healthineers, Memphis, TN). Initially, 1487 images were included. After excluding PET/CT image pairs with mismatches, that is 947 image pairs (~64%), 540 cases remained for 5-fold training. The semidiagnostic CTAC images acquired with an average tube current of ~110 mAs, and PET-NC and PET-ASC images were reconstructed using iterative reconstruction methods. Detailed information about dataset 1 can be found in Table 1. A total number of 22 organs were selected for tasks 1 and 2 on dataset 1, including the adrenal gland (AG), aorta, colon, esophagus, eyeballs, femoral heads (FHs), gall bladder (GB), heart, hip bones (including ilium, ischium, and pubis as a single mask), kidneys, liver, lungs, pancreas, erectus spinae, rib cage, sacrum, spleen, stomach, urinary bladder (UB), vertebrae, brain, and clavicle.

**TABLE 1:** Detailed Demographics of All 1487 Images Included as Dataset 1 (^18^F-FDG)

Scanner	Siemens Biograph mCT	Siemens Biograph Vision
Number	689	798
kVp	80, 100, 110, 140	80, 100, 110, 140
CTDI_vol_ (mGy)	3.92 ± 2.29 (0.25–20.85)	4.49 ± 2.24 (0.63–24.16)
Age (y)	61.639 ± 16.166 (5.0–93.0)	61.265 ± 16.993 (6.0–96.0)
Gender	Female: 376, male: 313	Female: 439, male: 359
Date	2014–2021	2019–2021
Patient height (m)	1.66 ± 0.14 (1.34–1.98)	1.68 ± 0.10 (0.72–2.0)
Patient weight (kg)	69.1 ± 16.6 (29.4–164)	70.1 ± 16.4 (21.3–151.0)
Average tube current (mA)	129.5 ± 51.5 (24.3–314.1)	128.1 ± 48.1 (32.0–476.421)
PET reconstruction	OSEM 3D + PSF + TOF	OSEM 3D + PSF + TOF

#### Dataset 2 (^68^Ga-PSMA)

These data included a total number of 575 PET/CT images injected with ^68^Ga-PSMA radiopharmaceutical scanned on 4 different Siemens scanners at 3 different nuclear medicine centers. All patients were male.

From the 575 cases initially collected, 390 were excluded, leaving only 185 clean cases for the remainder of our study. Table 2 summarizes the demographic information for all 575 cases initially included in our study. Some information was missing due to the anonymization process. A total number of 15 organs were selected for tasks 3 and 4 on dataset 2, including AG aorta, brain, eyeballs, hip bones, kidneys, liver, lungs, pancreas, rib cage, sacrum, spleen, UB, vertebrae, and heart. Seven organs were excluded from dataset 2 for tasks 3 and 4 as there is less anatomical information in ^68^Ga images compared with ^18^F-FDG images. We aimed to include only organs with distinguishable uptake.

**TABLE 2:** Demographic Information of All Included 575 ^68^Ga-PET/CT Images From Dataset 2 in This Study

Scanner	SIEMENS Biograph	SIEMENS Biograph Horizon	SIEMENS Biograph 128 Vision	SIEMENS Biograph 128 mCT
No. images	314	69	112	80
Age (y)	67.3 ± 8.2 (40–93)	65.3 ± 10.9 (17–88)	71.1 ± 8.1 (50–90)	71.3 ± 7.8 (54–93)
Date	2016 to 2020	2021 to 2022	2019 to 2021	2019 to 2021
Center	1	2	3	4
kVp	N/A	110 and 130	100, 120, 140	100, 120, 140
Pitch	N/A	1.2 ± 0.0 (1.2–1.2)	0.8 ± 0.0 (0.8–0.8)	0.8 ± 0.0 (0.8–0.8)
CTDI_vol_ (mGy)	N/A	4.21 ± 1.78 (1.94–11.91)	5.16 ± 1.84 (0.66–14.14)	5.10 ± 1.75 (1.18–12.17)
Average tube current (mA)	93.4 ± 13.4 (44.5–122.9)	N/A	117.8 ± 26.1 (73.3–264.2)	121.6 ± 31.2 (76.7–318.1)
Time per bed (s)	180 ± 24 (220–270)	87 ± 29 (60–120)	250 ± 18 (142–266)	212 ± 10 (15–217)

### Model Training Parameters

Four separated nnU-Net^40^ models were trained for the 4 defined tasks. For each task, the combined segmentation masks and the corresponding PET images were fed into an nnU-Net version 2 (nnunetv2) pipeline using default parameters except the training length, which was increased from the default value of 1000 epochs to 2000 epochs to enhance accuracy. We utilized nnU-Net 3D-fullres training configuration, which uses 3D patches for training. The initial learning rate was set to 1e-2 and decreased every epoch. The decay of 3e-5 and the Dice cross-entropy loss function were used. Five-fold cross-validation data splits were used, with 80% of images used for training and 20% for testing in each fold. The training process was conducted on a PC equipped with an RTX4090 GPU with 24 GB of dedicated memory and a Core i9-13900KF CPU with 32 GB of RAM.

### Evaluation Strategy

Common segmentation evaluation metrics, including Dice coefficient, Jaccard index, precision, sensitivity, specificity, accuracy, mean surface distance, and segment volume difference, were used to compare the predicted segmentation with the reference ones. The Mann-Whitney *U* test was employed to compare the models’ performance on NC and ASC images. In other words, we compared performance between tasks 1 and 2 as well as between tasks 3 and 4, seeking statistically significant differences using a 2-tailed *P* value of 0.05 as the threshold.

In summary, the proposed methodology includes:

1. Generating segmentations on CT images.
2. Visual assessment and excluding cases presenting with PET and CT misalignment.
3. Resampling and converting the segmentation outputs to PET SUV images.
4. Training the modified nnU-Net segmentation models for each task for 2 tracers, namely, ^18^F-FDG and ^68^Ga-PSMA.

Finally, the models’ performance was evaluated for each task.

## RESULTS

### Tasks 1 and 2

**FIGURE 3:**
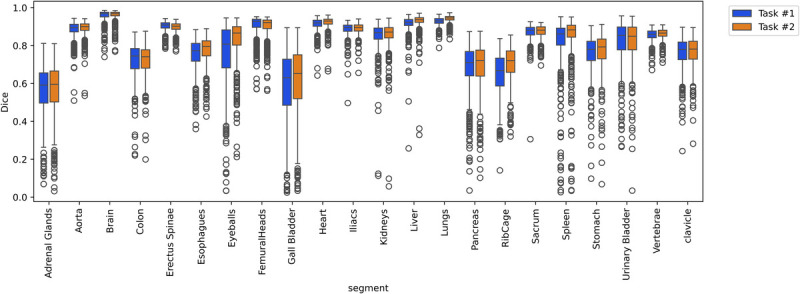
Box plot of Dice coefficients for task 1 versus 2.

For tasks 1 and 2, an average Dice value over all organs of 0.81 ± 0.15 and 0.82 ± 0.14 was achieved, respectively. As shown in Figure 3, in terms of Dice scores, task 1 demonstrated superior performance across most organs compared with task 2.

Tables 3 and 4 summarize the details of 5-fold cross-validation results for tasks 1 and 2, respectively. The highest Dice coefficients were achieved for the brain and lungs, whereas the lowest values were found for smaller organs, such as AGs. The detailed results separated by every fold may be found in Supplementary Table 1, http://links.lww.com/CNM/A531. Supplementary Figure 1 compares the resulting Dice for all tasks, http://links.lww.com/CNM/A531.

**TABLE 3:** Average Performance Metrics of Our Models for Task 1 From 5-Fold Cross-Validation

Task	Segment	Dice	Jaccard	Sensitivity	Specificity	Precision	Accuracy	Mean Surface Distance (mm)	Volume Difference (mL)
Task 1	AG	0.561 ± 0.135	0.402 ± 0.122	0.572 ± 0.157	1.0 ± 0.0	0.579 ± 0.146	1.0 ± 0.0	2.231 ± 2.006	−0.309 ± 2.588
	Aorta	0.884 ± 0.045	0.796 ± 0.066	0.888 ± 0.051	1.0 ± 0.0	0.883 ± 0.054	1.0 ± 0.0	1.023 ± 0.629	−0.52 ± 26.031
	Brain	0.958 ± 0.032	0.92 ± 0.054	0.956 ± 0.032	1.0 ± 0.0	0.96 ± 0.041	1.0 ± 0.0	6.631 ± 14.308	−7.448 ± 64.478
	Clavicles	0.762 ± 0.084	0.622 ± 0.101	0.767 ± 0.092	1.0 ± 0.0	0.761 ± 0.094	1.0 ± 0.0	2.529 ± 4.705	−0.055 ± 9.39
	Colon	0.725 ± 0.091	0.576 ± 0.103	0.711 ± 0.1	1.0 ± 0.0	0.745 ± 0.095	0.999 ± 0.0	4.062 ± 2.404	−48.169 ± 141.816
	Esophagus	0.754 ± 0.085	0.612 ± 0.101	0.763 ± 0.096	1.0 ± 0.0	0.751 ± 0.094	1.0 ± 0.0	1.295 ± 0.857	0.528 ± 7.615
	Eyeballs	0.757 ± 0.171	0.635 ± 0.192	0.779 ± 0.171	1.0 ± 0.0	0.742 ± 0.177	1.0 ± 0.0	1.879 ± 2.475	1.14 ± 2.745
	FHs	0.905 ± 0.058	0.831 ± 0.086	0.914 ± 0.047	1.0 ± 0.0	0.9 ± 0.079	1.0 ± 0.0	3.607 ± 9.742	22.545 ± 99.621
	GB	0.586 ± 0.193	0.439 ± 0.182	0.596 ± 0.21	1.0 ± 0.0	0.625 ± 0.214	1.0 ± 0.0	5.102 ± 8.522	−2.832 ± 14.219
	Heart	0.913 ± 0.034	0.841 ± 0.053	0.918 ± 0.049	1.0 ± 0.0	0.911 ± 0.048	1.0 ± 0.0	3.038 ± 5.685	3.421 ± 64.693
	Hips	0.887 ± 0.036	0.799 ± 0.053	0.886 ± 0.039	1.0 ± 0.0	0.889 ± 0.045	1.0 ± 0.0	0.809 ± 0.386	−4.637 ± 34.58
	Kidneys	0.851 ± 0.077	0.747 ± 0.095	0.866 ± 0.084	1.0 ± 0.0	0.844 ± 0.087	1.0 ± 0.0	2.164 ± 3.047	5.254 ± 49.649
	Liver	0.915 ± 0.049	0.846 ± 0.067	0.92 ± 0.063	1.0 ± 0.0	0.914 ± 0.052	1.0 ± 0.0	2.48 ± 1.942	3.991 ± 222.317
	Lungs	0.927 ± 0.022	0.865 ± 0.037	0.929 ± 0.034	1.0 ± 0.0	0.927 ± 0.038	0.999 ± 0.0	1.824 ± 0.841	4.763 ± 214.312
	Pancreas	0.682 ± 0.126	0.53 ± 0.131	0.688 ± 0.143	1.0 ± 0.0	0.691 ± 0.131	1.0 ± 0.0	3.561 ± 3.175	−1.579 ± 19.486
	RAM	0.903 ± 0.025	0.824 ± 0.041	0.902 ± 0.03	1.0 ± 0.0	0.906 ± 0.036	1.0 ± 0.0	1.083 ± 0.387	−9.367 ± 54.561
	Rib cage	0.651 ± 0.111	0.492 ± 0.116	0.662 ± 0.115	1.0 ± 0.0	0.645 ± 0.12	0.999 ± 0.0	1.729 ± 1.758	10.792 ± 68.369
	Sacrum	0.872 ± 0.042	0.776 ± 0.056	0.888 ± 0.046	1.0 ± 0.0	0.859 ± 0.05	1.0 ± 0.0	1.237 ± 0.791	8.624 ± 16.751
	Spleen	0.819 ± 0.141	0.711 ± 0.153	0.834 ± 0.148	1.0 ± 0.0	0.82 ± 0.142	1.0 ± 0.0	3.581 ± 6.563	4.592 ± 58.892
	Stomach	0.758 ± 0.1	0.62 ± 0.113	0.763 ± 0.118	1.0 ± 0.0	0.767 ± 0.106	1.0 ± 0.0	3.637 ± 3.425	−3.837 ± 43.979
	UB	0.821 ± 0.112	0.71 ± 0.141	0.832 ± 0.137	1.0 ± 0.0	0.835 ± 0.138	1.0 ± 0.0	3.774 ± 15.654	−4.205 ± 44.513
	Vertebrae	0.856 ± 0.031	0.75 ± 0.046	0.862 ± 0.035	1.0 ± 0.0	0.852 ± 0.04	1.0 ± 0.0	1.306 ± 2.694	11.017 ± 53.781

**TABLE 4:** Evaluation Metrics for Task 2, Averaged Over 5-Fold Cross-Validation

Task	Segment	Dice	Jaccard	Sensitivity	Specificity	Precision	Accuracy	Mean Surface Distance (mm)	Volume Difference (mL)
Task 2	AG	0.568 ± 0.139	0.409 ± 0.126	0.594 ± 0.156	1.0 ± 0.0	0.569 ± 0.149	1.0 ± 0.0	2.31 ± 2.474	0.272 ± 2.635
	Aorta	0.892 ± 0.039	0.807 ± 0.057	0.892 ± 0.046	1.0 ± 0.0	0.895 ± 0.046	1.0 ± 0.0	0.943 ± 0.61	−2.592 ± 24.324
	Brain	0.961 ± 0.027	0.925 ± 0.045	0.961 ± 0.027	1.0 ± 0.0	0.962 ± 0.039	1.0 ± 0.0	7.414 ± 18.699	−2.775 ± 69.11
	Clavicles	0.767 ± 0.079	0.628 ± 0.097	0.773 ± 0.089	1.0 ± 0.0	0.765 ± 0.089	1.0 ± 0.0	2.974 ± 5.793	0.244 ± 9.83
	Colon	0.723 ± 0.083	0.573 ± 0.095	0.717 ± 0.092	1.0 ± 0.0	0.736 ± 0.094	0.999 ± 0.0	4.169 ± 2.304	−28.668 ± 128.858
	Esophagus	0.777 ± 0.072	0.641 ± 0.09	0.791 ± 0.084	1.0 ± 0.0	0.768 ± 0.083	1.0 ± 0.0	1.261 ± 2.314	1.269 ± 6.881
	Eyeballs	0.832 ± 0.108	0.725 ± 0.135	0.836 ± 0.105	1.0 ± 0.0	0.836 ± 0.122	1.0 ± 0.0	1.974 ± 12.072	−0.064 ± 2.837
	FH	0.899 ± 0.058	0.822 ± 0.086	0.906 ± 0.047	1.0 ± 0.0	0.896 ± 0.079	1.0 ± 0.0	3.741 ± 9.495	19.62 ± 96.896
	GB	0.607 ± 0.197	0.461 ± 0.185	0.627 ± 0.207	1.0 ± 0.0	0.63 ± 0.219	1.0 ± 0.0	5.11 ± 8.44	−1.745 ± 14.354
	Heart	0.922 ± 0.03	0.857 ± 0.047	0.928 ± 0.042	1.0 ± 0.0	0.919 ± 0.04	1.0 ± 0.0	2.799 ± 5.65	5.597 ± 52.358
	Hips	0.889 ± 0.031	0.802 ± 0.047	0.888 ± 0.035	1.0 ± 0.0	0.892 ± 0.04	1.0 ± 0.0	0.815 ± 0.461	−5.193 ± 36.071
	Kidneys	0.856 ± 0.075	0.754 ± 0.091	0.874 ± 0.078	1.0 ± 0.0	0.845 ± 0.086	1.0 ± 0.0	2.078 ± 2.702	9.694 ± 42.382
	Liver	0.929 ± 0.046	0.869 ± 0.061	0.937 ± 0.058	1.0 ± 0.0	0.924 ± 0.043	1.0 ± 0.0	2.139 ± 1.989	16.911 ± 199.752
	Lungs	0.943 ± 0.018	0.892 ± 0.031	0.935 ± 0.027	1.0 ± 0.0	0.952 ± 0.026	0.999 ± 0.0	1.456 ± 0.806	−64.043 ± 153.441
	Pancreas	0.692 ± 0.122	0.541 ± 0.128	0.707 ± 0.137	1.0 ± 0.0	0.692 ± 0.128	1.0 ± 0.0	3.543 ± 3.336	0.945 ± 20.422
	RAM	0.897 ± 0.025	0.814 ± 0.039	0.89 ± 0.029	1.0 ± 0.0	0.906 ± 0.036	1.0 ± 0.0	1.201 ± 0.504	−23.925 ± 57.449
	Rib cage	0.704 ± 0.093	0.55 ± 0.103	0.703 ± 0.097	1.0 ± 0.0	0.708 ± 0.102	0.999 ± 0.0	1.486 ± 1.755	−8.441 ± 63.949
	Sacrum	0.875 ± 0.033	0.779 ± 0.051	0.888 ± 0.04	1.0 ± 0.0	0.864 ± 0.044	1.0 ± 0.0	1.243 ± 0.994	7.263 ± 17.211
	Stomach	0.769 ± 0.101	0.634 ± 0.116	0.771 ± 0.121	1.0 ± 0.0	0.779 ± 0.106	1.0 ± 0.0	3.545 ± 3.688	−4.948 ± 42.206
	Spleen	0.847 ± 0.129	0.751 ± 0.143	0.859 ± 0.132	1.0 ± 0.0	0.849 ± 0.128	1.0 ± 0.0	3.265 ± 6.425	3.099 ± 60.25
	UB	0.821 ± 0.109	0.709 ± 0.137	0.836 ± 0.132	1.0 ± 0.0	0.83 ± 0.139	1.0 ± 0.0	4.263 ± 16.277	−2.777 ± 45.19
	Vertebrae	0.862 ± 0.028	0.759 ± 0.042	0.866 ± 0.032	1.0 ± 0.0	0.86 ± 0.037	1.0 ± 0.0	1.253 ± 2.744	6.596 ± 51.721

Table 5 compares *P* values between tasks 1 and 2, indicating significant differences across most organs. There are significant differences in Dice values for most organs between tasks 1 and 2, whereas volume differences show significance in fewer organs. It should be noted that there was statistically significant difference between tasks 1 and 2 for some organs, but the difference between averages is negligible. An example of our model output for task 2 tested on a noisy dynamic acquisition is shown in Supplementary Figure 2, http://links.lww.com/CNM/A531.

**TABLE 5:** Mann-Whitney *P* Values Comparing Task 1 and Task 2

Organ	Dice	Jaccard	Mean Surface Distance	Volume Difference
Liver	0.000	0.000	0.000	0.089
Brain	0.619	0.619	0.485	0.164
AG	0.236	0.236	0.315	0.001
Stomach	0.011	0.011	0.069	0.473
Rib cage	0.000	0.000	0.000	0.000
Colon	0.396	0.396	0.136	0.012
Erectus spinae	0.000	0.000	0.000	0.000
Sacrum	0.318	0.318	0.248	0.255
Aorta	0.003	0.003	0.003	0.024
Clavicle	0.385	0.385	0.887	0.633
Esophagus	0.000	0.000	0.000	0.068
Vertebrae	0.001	0.001	0.006	0.092
Eyeballs	0.000	0.000	0.000	0.000
FH	0.000	0.000	0.004	0.342
GB	0.034	0.034	0.261	0.061
Spleen	0.000	0.000	0.000	0.458
Kidneys	0.162	0.162	0.296	0.263
Lungs	0.000	0.000	0.000	0.000
Hips	0.421	0.421	0.763	0.855
Pancreas	0.175	0.175	0.350	0.022
UB	0.741	0.741	0.362	0.463
Heart	0.000	0.000	0.000	0.907

*P* < 0.05 reflects statistically significant difference.

**FIGURE 4:**
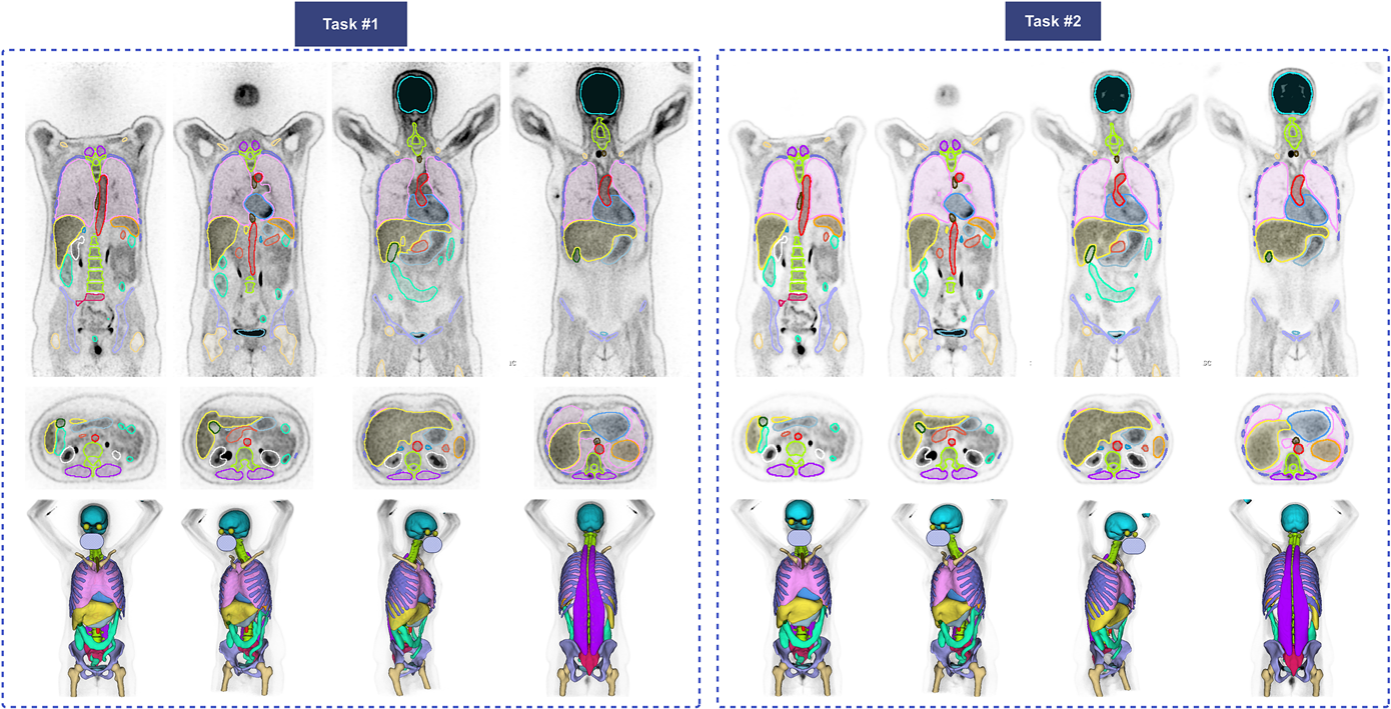
Coronal (top), axial (middle), and 3D (bottom) visualizations of the segmentations for tasks 1 and 2. Each color presents 1 organ; the internal organs, such as kidneys, are not visible in 3D-rendered images. The face is masked for privacy.

Figure 4 demonstrates an example of segmented organs for tasks 1 and 2 on a case with a good match between PET and CT images from a cross-validation strategy, depicting the excellent performance of our models.

**FIGURE 5:**
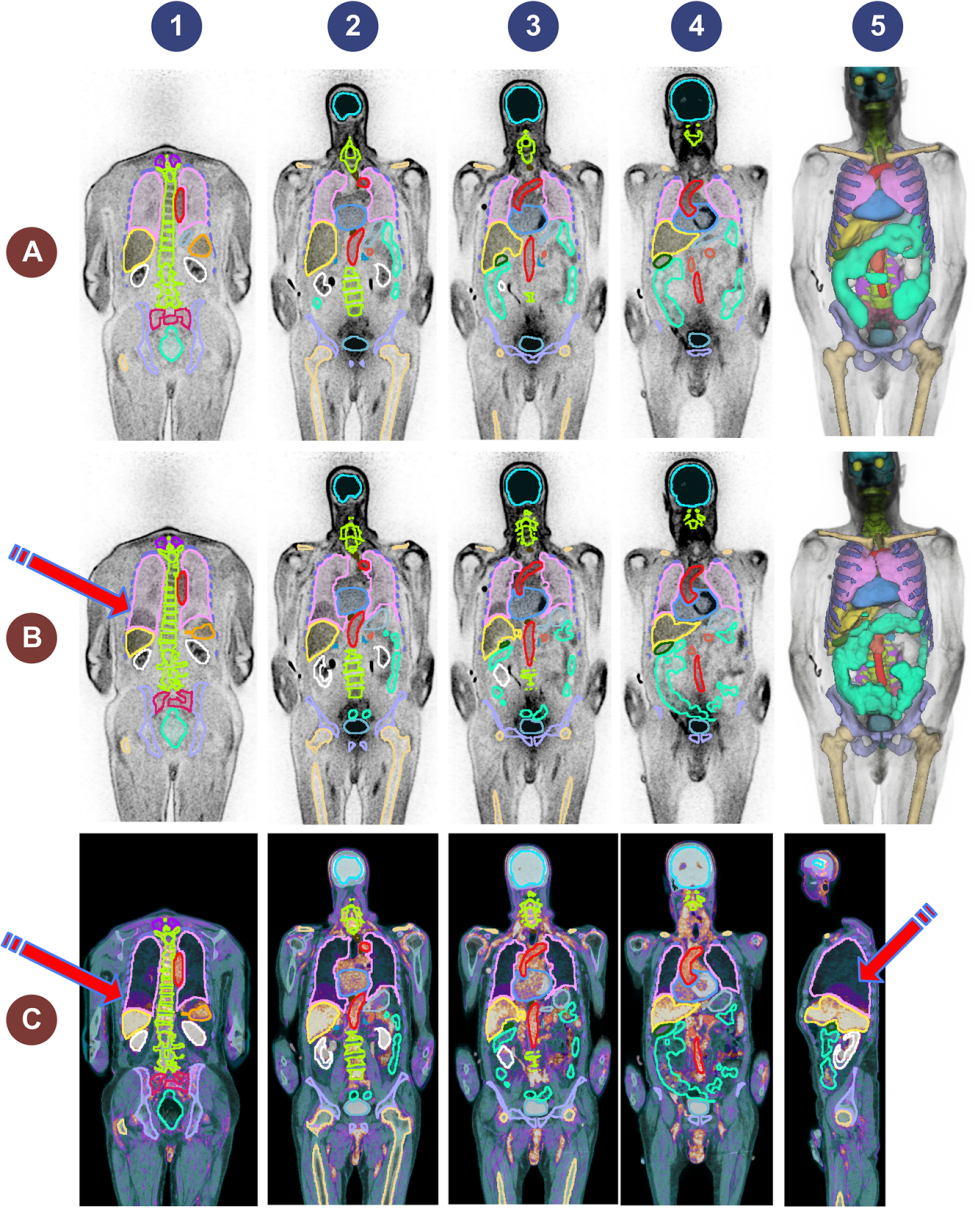
Representative excluded case presenting an image with respiratory mismatch between PET and CT images. **A** (top row), PET-NC image and task 1 generated masks, columns 1 to 4 show coronal images, whereas column 5 shows the 3D-rendered segmentations. **B** (middle row), PET-NC image and the segmentation masks generated on the coregistered CTAC image, columns 1 to 4 show coronal images, whereas column 5 shows the 3D-rendered segmentations. The arrow shows the mismatch at lung/liver interface. **C** (bottom row), The fused PET-NC and CT in coronal (columns 1 to 4) and sagittal (column 5) views. The arrows highlight the mismatch regions.

Figure 5 shows an example of PET/CT image with unreliable CT segmentation due to respiratory mismatch affecting the segmentation of moving organs, especially the lungs, liver, and spleen. This case was excluded from cross-validation training; the trained models of tasks 1 and 2 were ensembled on the corresponding images. In other words, task 1 model was tested on PET-NC and task 2 model on PET-ASC images.

### Tasks 3 and 4

**FIGURE 6:**
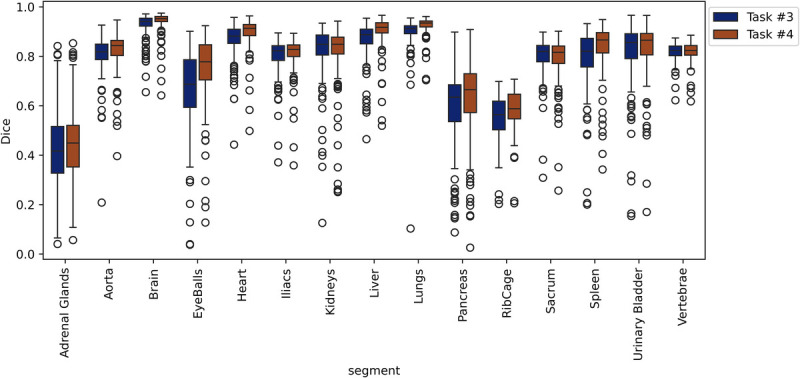
Box plots showing the Dice scores for task 3 and 4 for every 15 included organs.

The averages of the Dice scores over 15 organs from 5-fold cross-validation were 0.766 ± 0.171 and 0.788 ± 0.163 for tasks 3 and 4, respectively. The performance metrics for tasks 3 and 4 are summarized in Tables 6 and 7. The detailed performance metrics are reported separately for each fold in Supplementary Table 1, http://links.lww.com/CNM/A531. The Dice values for task 4 were significantly higher than those for task 3 with *P* values below 0.05 for most organs, except for larger organs with a clear objective contrast on ^68^Ga-NC images, such as the hips, sacrum, vertebrae, kidneys, and UB. The *P* values are reported in Table 8. The lowest Dice value was observed for AG, whereas the highest was achieved for the brain. Figure 6 displays the box plot of Dice scores for the included organs in tasks 3 and 4.

**TABLE 6:** Average Segmentation Metrics From 5-Fold Cross-Validation for Task 3 for All Included Organs

Task	Segment	Dice	Jaccard	Sensitivity	Specificity	Precision	Accuracy	Mean Surface Distance (mm)	Volume Difference (mL)
Task 3	AG	0.421 ± 0.154	0.279 ± 0.13	0.391 ± 0.154	1.0 ± 0.0	0.482 ± 0.179	1.0 ± 0.0	4.085 ± 4.066	−2.404 ± 3.93
	Aorta	0.809 ± 0.076	0.685 ± 0.092	0.806 ± 0.091	1.0 ± 0.0	0.819 ± 0.074	0.999 ± 0.0	2.264 ± 4.585	−9.15 ± 41.849
	Brain	0.928 ± 0.046	0.868 ± 0.072	0.924 ± 0.057	1.0 ± 0.0	0.934 ± 0.051	0.999 ± 0.001	5.735 ± 10.507	−16.978 ± 83.016
	Eyeballs	0.67 ± 0.163	0.524 ± 0.169	0.678 ± 0.169	1.0 ± 0.0	0.669 ± 0.168	1.0 ± 0.0	4.195 ± 23.087	0.403 ± 4.944
	Heart	0.869 ± 0.065	0.774 ± 0.09	0.871 ± 0.081	1.0 ± 0.0	0.872 ± 0.073	0.999 ± 0.0	6.668 ± 17.124	−6.179 ± 86.089
	Hips	0.804 ± 0.069	0.677 ± 0.084	0.804 ± 0.079	0.999 ± 0.0	0.806 ± 0.072	0.999 ± 0.0	2.324 ± 5.632	−6.756 ± 68.178
	Kidneys	0.824 ± 0.109	0.712 ± 0.129	0.83 ± 0.119	1.0 ± 0.0	0.827 ± 0.104	0.999 ± 0.0	2.837 ± 4.473	1.095 ± 79.212
	Liver	0.867 ± 0.075	0.772 ± 0.103	0.874 ± 0.088	0.999 ± 0.001	0.866 ± 0.081	0.998 ± 0.001	4.052 ± 3.702	5.234 ± 241.476
	Lungs	0.899 ± 0.071	0.821 ± 0.081	0.899 ± 0.083	0.999 ± 0.001	0.906 ± 0.048	0.997 ± 0.001	2.578 ± 2.813	−25.67 ± 344.082
	Pancreas	0.596 ± 0.159	0.441 ± 0.152	0.575 ± 0.175	1.0 ± 0.0	0.645 ± 0.171	1.0 ± 0.0	5.236 ± 6.45	−13.614 ± 30.184
	Rib cage	0.555 ± 0.085	0.389 ± 0.078	0.575 ± 0.091	0.999 ± 0.0	0.542 ± 0.095	0.997 ± 0.001	2.791 ± 1.588	47.523 ± 118.76
	Sacrum	0.802 ± 0.072	0.675 ± 0.086	0.817 ± 0.083	1.0 ± 0.0	0.792 ± 0.077	1.0 ± 0.0	2.492 ± 1.386	8.747 ± 30.328
	Spleen	0.794 ± 0.119	0.671 ± 0.14	0.792 ± 0.125	1.0 ± 0.0	0.808 ± 0.135	1.0 ± 0.0	3.542 ± 4.103	−7.187 ± 39.075
	UB	0.822 ± 0.126	0.713 ± 0.147	0.818 ± 0.147	1.0 ± 0.0	0.848 ± 0.136	1.0 ± 0.0	3.83 ± 9.019	−9.085 ± 49.223
	Vertebrae	0.818 ± 0.036	0.694 ± 0.049	0.826 ± 0.043	0.999 ± 0.0	0.812 ± 0.046	0.998 ± 0.0	1.527 ± 0.483	18.014 ± 83.173

**TABLE 7:** Performance Metrics From 5-Fold Cross-Validation for Task 4

Task	Segment	Dice	Jaccard	Sensitivity	Specificity	Precision	Accuracy	Mean Surface Distance (mm)	Volume Difference (mL)
Task 4	AG	0.437 ± 0.155	0.293 ± 0.133	0.414 ± 0.154	1.0 ± 0.0	0.485 ± 0.179	1.0 ± 0.0	3.355 ± 2.967	−1.98 ± 3.422
	Aorta	0.825 ± 0.075	0.708 ± 0.096	0.824 ± 0.081	1.0 ± 0.0	0.832 ± 0.085	1.0 ± 0.0	1.913 ± 2.273	−3.939 ± 69.773
	Brain	0.942 ± 0.042	0.893 ± 0.064	0.942 ± 0.053	1.0 ± 0.0	0.945 ± 0.044	0.999 ± 0.0	5.559 ± 11.292	−9.794 ± 84.242
	Eyeballs	0.753 ± 0.134	0.62 ± 0.151	0.747 ± 0.132	1.0 ± 0.0	0.774 ± 0.14	1.0 ± 0.0	6.797 ± 37.747	0.379 ± 15.853
	Heart	0.897 ± 0.057	0.818 ± 0.081	0.898 ± 0.073	1.0 ± 0.0	0.9 ± 0.061	0.999 ± 0.0	6.186 ± 17.138	−5.302 ± 73.578
	Hips	0.812 ± 0.068	0.688 ± 0.082	0.803 ± 0.08	0.999 ± 0.0	0.824 ± 0.067	0.999 ± 0.0	2.189 ± 6.148	−25.21 ± 70.011
	Kidneys	0.819 ± 0.12	0.707 ± 0.136	0.824 ± 0.118	1.0 ± 0.0	0.826 ± 0.126	0.999 ± 0.001	3.641 ± 8.759	5.333 ± 132.237
	Liver	0.904 ± 0.062	0.829 ± 0.087	0.906 ± 0.082	0.999 ± 0.0	0.906 ± 0.055	0.999 ± 0.001	2.599 ± 2.166	−9.704 ± 222.004
	Lungs	0.926 ± 0.035	0.864 ± 0.054	0.926 ± 0.047	0.999 ± 0.001	0.927 ± 0.037	0.998 ± 0.001	1.744 ± 1.395	−18.3 ± 226.443
	Pancreas	0.63 ± 0.151	0.476 ± 0.148	0.617 ± 0.164	1.0 ± 0.0	0.665 ± 0.17	1.0 ± 0.0	4.417 ± 5.866	−9.752 ± 28.548
	Rib cage	0.585 ± 0.077	0.417 ± 0.072	0.607 ± 0.081	0.999 ± 0.0	0.571 ± 0.092	0.997 ± 0.001	2.391 ± 1.414	49.412 ± 122.563
	Sacrum	0.793 ± 0.086	0.664 ± 0.101	0.804 ± 0.095	1.0 ± 0.0	0.786 ± 0.091	1.0 ± 0.0	3.595 ± 10.435	6.662 ± 33.149
	Spleen	0.839 ± 0.097	0.732 ± 0.123	0.838 ± 0.107	1.0 ± 0.0	0.847 ± 0.106	1.0 ± 0.0	2.752 ± 5.183	−2.885 ± 31.768
	UB	0.834 ± 0.11	0.728 ± 0.135	0.828 ± 0.138	1.0 ± 0.0	0.861 ± 0.125	1.0 ± 0.0	2.628 ± 2.176	−9.362 ± 46.993
	Vertebrae	0.819 ± 0.039	0.696 ± 0.053	0.831 ± 0.05	0.999 ± 0.0	0.81 ± 0.048	0.998 ± 0.001	1.54 ± 0.598	29.778 ± 96.0

**TABLE 8:** *P* Values of Mann-Whitney Statistical Test Comparing the Performance Metrics for Task 3 vs Task 4

Organ	*P*			
	Dice	Jaccard	Mean Surface Distance	Volume Difference
AG	0.269	0.269	0.049	0.210
Aorta	0.000	0.000	0.001	0.962
Brain	0.000	0.000	0.015	0.083
Eyeballs	0.000	0.000	0.000	0.000
Hips	0.141	0.141	0.054	0.004
Kidneys	0.668	0.668	0.776	0.373
Liver	0.000	0.000	0.000	0.359
Lungs	0.000	0.000	0.000	0.684
Pancreas	0.012	0.012	0.004	0.331
Rib cage	0.000	0.000	0.000	0.909
Sacrum	0.565	0.565	0.532	0.375
Spleen	0.000	0.000	0.000	0.607
UB	0.359	0.359	0.135	0.749
Vertebrae	0.684	0.684	0.837	0.164
Heart	0.000	0.000	0.000	0.979

**FIGURE 7:**
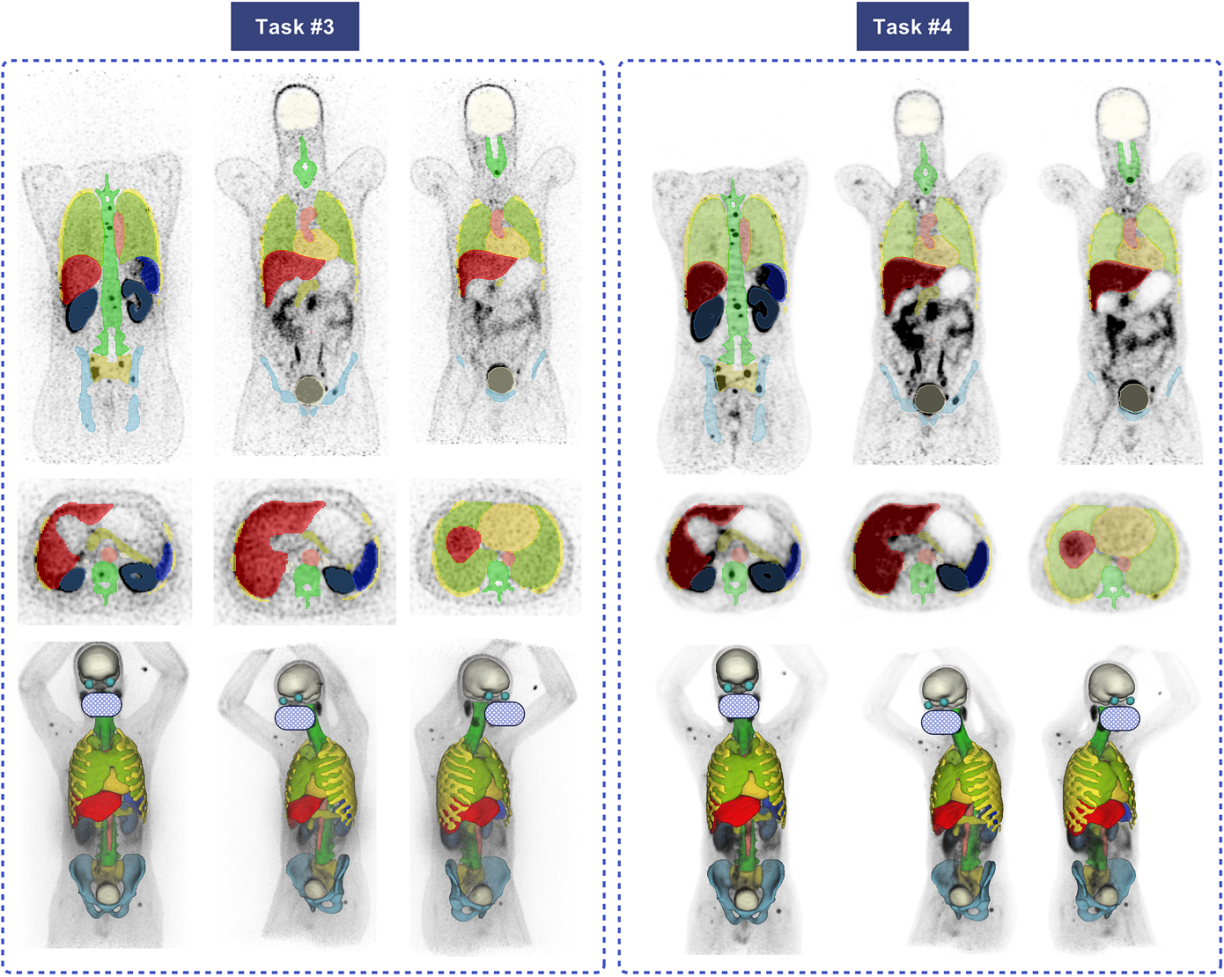
Representative case with segmentations generated on PET-NC (task 3) and PET-ASC images (task 4). Top row: coronal slices, middle row: axial, and bottom row: 3D-rendered segmentations. Face is masked for privacy.

**FIGURE 8:**
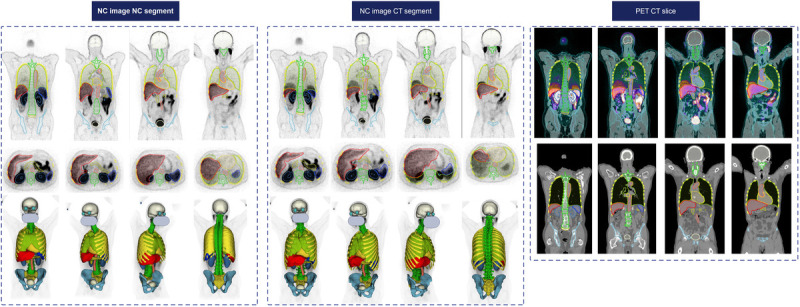
^68^Ga-PSMA PET/CT image with respiratory mismatch. It should be noted that the kidney boundaries are expanded in the visualized images due to selected window width/level. Face is masked for privacy.

Figure 7 illustrates an example with strong alignment between CT and PET within the 5-fold cross-validation data split for tasks 3 and 4. Figure 8 depicts an image from the excluded studies demonstrating the mismatch between PET and CT images. This case shows the unreliable CT generated masks and the excellent performance of our model in delineating organs.

## DISCUSSION

Automatic, fast, and accurate segmentation of medical images has become 1 of the hottest topics in precision medicine for personalized dosimetry and image quantification.^41–45^ In nuclear medicine, where PET/CT and SPECT/CT are commonly performed for diagnostic and therapeutic purposes, automated organ segmentation is crucial. Recent studies showed the importance of organomics and organ information in overall survival prediction^15,20^ as well as misalignment detection on PET/CT images,^46^ as well as pretherapy dose prediction in PSMA theragnostic procedures.^14^ The common method used for automated organ delineation in hybrid PET/CT and SPECT/CT relies on the coregistered CT images. However, this approach is subject to limitations, such as the highly prevalent mismatch between emission and CT images and the low quality of low-dose CTAC images. UB filling and bowel movements are inevitable and could cause more problematic mismatch between the CT generated masks and realistic organ position, shape, and size depicted on PET images. Additionally, not all PET scanners are equipped with CT for attenuation and scatter correction, and as such, an approach that does not rely on CT is necessary.

We targeted 2 commonly used tracers for PET imaging and developed a comprehensive DL segmentation pipeline for the automated delineation of multiple organs to be used in different clinical scenarios. To ensure a strong match between emission and CT images in the training set and to prevent the trained DL models from being affected by mismatch, we first cleaned our data by excluding PET/CT image pairs presenting with respiratory, cardiac, and bulk motion mismatches. After visual assessment of our initially included dataset, we excluded more than 65% of the 2062 images from our study, emphasizing the high prevalence of mismatch in PET/CT imaging.

We included different numbers of organs depending on the tracer as ^68^Ga-PSMA PET images contain less anatomical information. Our goal was to develop a reliable model based on clinical studies for potential implementation in clinical setting. The first scenario for using our models involve performing segmentation on PET ASC images corrected either by CT or any other method, such as MLAA or DL-based ASC techniques. In this scenario, the CT image is either unavailable or too noisy to be segmented through DL. To address this limitation, we provided models from tasks 2 and 4 for delineation on PET ASC images, as they outperformed the models of tasks 1 and 3, which use NC images. PET ASC images benefit from better contrast and contain more information due to corrections for degrading factors, such as attenuation, scatter, and point-spread function (PSF).

Additionally, we considered the second scenario of performing CT-less PET segmentation where the PET ASC image is corrected with a mismatched CT, or PET-ASC images are not available. Such corrections can induce unacceptable mismatch artifacts on PET ASC images, removing the useful information, for example, in areas such as chest abdomen interval or causing halo artifacts which are very common in ^68^Ga-PSMA PET/CT imaging. In this scenario, as shown in Figure 5 and Figure 8, the chest area was affected, and the DL model trained on PET ASC images only on cases without mismatch may identify it as lung tissue. To address this issue, we implemented 2 strategies including tasks 1 and 3 to provide a reliable segmentation solution for all potential clinical scenarios. The performance of our models in the second scenario is lower than those in the first scenario as the NC images suffer from multiple artifacts, and are not corrected for attenuation and scatter, and usually do not include time-of-flight (TOF) and PSF correction. We hypothesize that this would be a versatile solution by considering real clinical needs and could tackle the issue more effectively. As presented in Supplementary Figure 3, http://links.lww.com/CNM/A531, there is minimal difference between SUV_mean_ calculated on task 1 and task 2 compared with reference segmentations, showing an excellent match on clinically relevant information extracted from generated masks by both tasks.

We employed state-of-the-art nnU-Net V2 pipeline, which has shown promising results in recent medical image segmentation studies. Our model achieved excellent accuracy in segmenting organs, such as the lungs, brain, and liver. However, it achieved lower performance in a few smaller organs with lower objective contrast and visibility in PET images, especially when using NC images as input, such as AGs. The overall performance was superior in tasks 1 and 2 using FDG PET images compared with tasks 3 and 4 using ^68^Ga-PET, as anticipated. The difference can be attributed to the lower structural information in ^68^Ga-PET images because of specific uptake patterns of ^68^Ga-PSMA tracer. As shown in Supplementary Figure 2, http://links.lww.com/CNM/A531, our models have shown acceptable performance even on very noisy dynamic FDG PET images. It should be noted that these images are acquired shortly after tracer injection, thus a different radiopharmaceutical uptake pattern can be observed compared with delayed PET images (usually acquired around 60 minutes postinjection). Despite this fact, our model showed robust performance on dynamic, noisy frames.

While the Dice coefficient alone may have limitations for evaluation of image segmentation performance,^47^ we extensively evaluated the performance of our models using multiple metrics, including Dice, Jaccard, mean surface distance, and the volume difference between the reference and the predicted masks. Our study achieved significantly better results compared with the study by Yazdani et al^36^ where few organs were included for segmentation. Our Dice scores were 0.82 versus 0.80, 0.90 versus 0.88, 0.84 versus 0.79, and 0.83 versus 0.81 for kidneys, liver, spleen, and UB organs in task 4, respectively. The improved performance could be due to our approach of excluding cases with mismatches, which could mislead the DL model during training and underestimate the Dice value when unreliable segmentation masks are used as reference in those cases. Klyuzhin et al^35^ developed a multiorgan segmentation model for ^68^Ga-PSMA images using both PET and CT images as inputs in their UNET model. Our model, however, utilizes only the emission images to overcome the aforementioned limitations. Organ segmentation directly on PET images ensures alignment between the segmentation masks and the actual metabolic uptake observed on the PET scan, thus eliminating the risk of misalignment. For example, in pharmacokinetic modeling from dynamic PET studies, the bladder fills progressively, and both the thoracic and abdominal organs move during the dynamic imaging procedure. Using segmentation that tracks these movements directly on PET image frames can prevent errors in parametric imaging caused by relying on a single CT-derived masks acquired either before or after dynamic PET imaging. Tracer uptake can also be misclassified between moving organs. For instance, for personalized dosimetry purposes, time-integrated activity is calculated by fitting a curve to the tracer uptake changes within each organ or VOI over time. Misalignment can result in the activity of organs, such as the lungs, spleen, liver, or colon being inaccurately recorded in adjacent structures, disrupting the curve-fitting process. Similarly, the UB, often empty in the initial frames when the CT scan is performed, fills during the dynamic acquisition. If the segmentation fails to account for this change, it may underestimate the tracer accumulation in the bladder and erroneously attribute it to remainder structures, leading to significant dose calculation errors. The inference time depends on the local machine performance. It was about 50 seconds per whole-body PET image on an NVIDIA RTX 4090 GPU requiring less than 7 GB of dedicated GPU RAM. We added some steps, such as cropping to the foreground to increase the inference speed. The code can be found on GitHub where we shared the models.

As presented in Supplementary Figure 4, http://links.lww.com/CNM/A531, the trained models in task 2 (trained on ^18^F-FDG images) can segment most organs accurately on ^68^Ga-PSMA images. Likewise, task 4 (trained on ^68^Ga images) models can delineate most of the organs on ^18^F-FDG images, especially bones, bladder, and lungs. Cross-tracer inference results suggest a great capacity for transfer learning on newly developed tracers or other available tracers. We made the models and training architecture publicly available to enable users developing new models by fine-tuning these models, the user can select a baseline model based on the similarities between the target tracer distribution and the pretrained models. However, developing a tracer-free model which may segment organs on any PET images is not recommended as we have a limited number of PET tracers and the injected tracer is usually known.

This work inherently bears a number of limitations. First, we developed PET organ segmentation models for 2 common tracers and suggested training new models for other tracers. However, transfer learning is 1 option that should be considered. CT segmentation does not share this dependency on the tracer used. Another limitation of our study is the limited number of training dataset acquired on only 2 PET/CT scanners from the same manufacturer. Potential end-users would need to perform fine-tuning on our publicly available models using their own local datasets.

## CONCLUSIONS

We developed DL-powered CT-less automated organ segmentation models from PET images for 2 common tracers used in PET/CT imaging to overcome the limitations of CT segmentation in delineation and quantification. Our model showed acceptable performance; however, the models using PET-ASC images as input achieved a better performance. The method can be used in the clinical to enable a number of clinical and research applications.
